# Regular Aerobic Voluntary Exercise Increased Oxytocin in Female Mice: The Cause of Decreased Anxiety and Increased Empathy-Like Behaviors

**DOI:** 10.4274/balkanmedj.galenos.2019.2018.12.87

**Published:** 2019-08-22

**Authors:** Oğuz Yüksel, Mehmet Ateş, Servet Kızıldağ, Zeynep Yüce, Başar Koç, Sevim Kandiş, Güven Güvendi, Aslı Karakılıç, Hikmet Gümüş, Nazan Uysal

**Affiliations:** 1 Department of Sports Medicine, Dokuz Eylül University School of Medicine, İzmir, Turkey; 2 College of Vocational School of Health Services, Dokuz Eylül University School of Medicine, İzmir, Turkey; 3 Department of Medical Biology and Genetics, Dokuz Eylül University School of Medicine, İzmir, Turkey; 4 Department of Physiology, Dokuz Eylül University School of Medicine, İzmir, Turkey; 5 Dokuz Eylül University School of Sport Sciences and Technology, İzmir, Turkey

**Keywords:** Anxiety, brain, empathy, exercise, gender, oxytocin

## Abstract

**Background:**

It is known that regular physical activity reduces anxiety. Low anxiety levels affect mood, emotions, and empathy. Oxytocin is a powerful hormone that regulates social interaction, sexual reproduction, maternal–infant bonding, milk release, empathy, and anxiety. Empathy is an important behavior in the living community; and also important for sportsmen during sportive competition and daily living life, because sportsmen are also role model of people.

**Aims:**

To investigate the effects of voluntary physical activity on oxytocin, anxiety, and empathy levels as well as the relationship between them.

**Study Design:**

Animal experiment.

**Methods:**

Male and female mice were made to exercise in running wheel cages for 6 weeks. Their empathy and anxiety levels were evaluated by using Helping Behavior test and elevated plus maze and open field test, respectively. And then the brain and blood oxytocin levels were measured.

**Results:**

Empathy-like behavior was improved in both genders of the exercise groups (door-opening time decreased in both genders of exercise groups, p for both=0.0001). As a response to exercise, both the brain and serum oxytocin levels increased in female mice (both of p=0.0001); however, in males, oxytocin levels increased in only the brain (p<0.05). Anxiety levels decreased in all the exercise groups (increased time spent in the middle area of open field test, both genders, p=0.002; increased time spent in the open arms of elevated plus maze test, females p=0.004, males p=0.0001). There was a strong negative correlation between serum oxytocin levels and door opening time of helping behavior equipment, and moderate negative correlation was found between the brain oxytocin levels and door-opening time of helping behavior equipment in females. However, there was no correlation between both the brain and serum oxytocin levels and empathy behavior in males. But there were very strong positive correlations between low anxiety indicators and both the brain and serum oxytocin levels in both the genders.

**Conclusion:**

Voluntary physical activity decreases anxiety and increases empathy-like behavior in mice; which is associated with increased oxytocin levels in female mice but not in male mice. Further research is required to investigate the mechanisms of exercise effect on anxiety and empathic brain pathways in males.

There is a substantial body of literature demonstrating that regular exercise and physical activity render many health benefits, including prevention and improvement of metabolic diseases, such as type 2 diabetes mellitus, metabolic syndrome, obesity, heart conditions, stroke, and arthritis. The benefits of physical activity are not only limited to physical conditions but also encompass our psychological well-being. Research has shown that regular physical activity has significant benefits in psychiatric patients, rendering it an effective therapeutic strategy (1). Our previous studies documenting correlations between exercise and changes in brain biochemistry also support the positive effect of physical activity on mental health (2,3).

Many studies have shown that oxytocin is a neuropeptide associated with emotional behavior, including empathy. It plays an important role in the development of social signals during evolution from lower vertebrates to higher primates (4). Oxytocin has been shown to support attachment, trust, empathy, generosity, and positive social participation following intranasal administration in humans (5). Expression levels of oxytocin in the brain as well as the neural architecture of empathy have both been observed to be altered by life experiences through epigenetic modifications (6). Animal and human studies have shown that exercise increases oxytocin levels. A ten-minute running exercise increased the level of salivary oxytocin (7). Increased oxytocin levels following exercise were reported in the hypothalamus, brainstem, and nucleus solitarius of rats (4,5).

Physical activity positively affects cognitive areas of the brain, such as the hippocampus, prefrontal cortex, and amygdala, by increasing the cognitive functions and lowering the anxiety and depression levels. Exercise improves one’s mood and supports emotional progress (8,9), which can also be observed as a reduction in anxiety-like behavior in rats following voluntary exercise (10). Anxiety has been shown to be positively associated with helping behavior and empathy (11).

Empathy is the recognition and internalization of someone else’s feelings, condition, or behavior. In 1934, Alfred Adler described empathy as “to see with the eyes of another, to hear with the ears of another, and to feel with the heart of another” (12). Empathy is important for the survival and maintenance of society by preventing aggression against others and establishing healthy communication among its members (13). Empathic behavior has been shown to increase activity in the temporo-parietal cortex, ventromedial prefrontal cortex, hippocampus, and amygdala in the brain. Empathy is observed in many animal species, such as humans, primates, and rodents (14).

Empathy is affected by many factors, such as stressful situations, pain, depression, and autism (11,15). We showed that empathy increased with low-intensity acute stress in our last experiment (16). Empathy is a significant variable in the effectiveness of sporting activities. The empathy skills of individuals are important in the team performance, especially during high-stress sporting events. In team sports, the empathy skills displayed by a team member toward his/her teammates, coach, and competitors can be an important factor in team spirit and success. It allows a player to anticipate behavior and outcomes in advance (17). On the other hand, the reverse relationship between sports/physical activity and empathy and the effects of physical activity on empathic behavior has not been reported. The present study aimed to investigate the effects of voluntary physical activity on oxytocin, anxiety, and empathy levels as well as the relationship between them.

## MATERIALS AND METHODS

**Figure 1:**
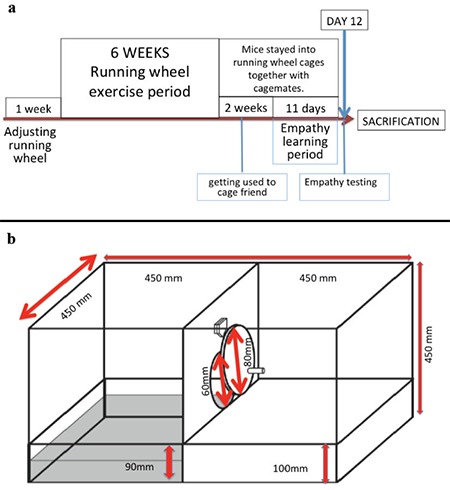
Timeline of the study (a), Empathy test equipment (b).

Thirty-two adult male and female Balb-c mice were used in the present study. With free access to laboratory chow and water, mice were housed in individual cages and kept in a 12h-light/12h-dark cycle at constant humidity (60%) and room temperature (22±1°C). Approval from Animal Care Committee of Dokuz Eylül University School of Medicine for the study was obtained. Mice were divided into four groups: (1) control females (n=8), (2) running wheel exercised females (n=8), (3) control males (n=8), and (4) running wheel exercised males (n=8). After an adaptation period of one week, a running wheel with a diameter of 11.5 cm was placed in the cage that the voluntary exercise group had free access to for 6 weeks (2). The mice were harbored in the same environment 14 days prior to the study for habituation; two mice were placed in each running wheel cage. Study design is shown in Figure 1a.

### Voluntary wheel running

Voluntary wheel running is a common model to investigate the physiological effects of aerobic exercise. The mice ran freely inside a plastic wheel. Daily running distance was calculated by the number of rotations of the wheel, counted by a digital counter connected to the wheel (18,19).

### Empathy-like Behavior test

All mice were trained via Helping Behavior test equipment (Figure 1b). Following the exercise period, all mice (including controls) were subjected to the Helping Behavior test during an 11-day training period. Previously described experimental equipment was used to test empathy-like behavior (16,20). Opening the door and saving the cage-mate was defined as empathy-like behavior. Mice were trained to open the door of the empathy test equipment for a total of 11 days and tested on the 12^th^ day. Each mouse assumed the roles of both the rescue mouse and the savior. Mice in pairs were subsequently placed in the platform’s rescue section and water section as shown in Figure 1b. Testing sessions were 5 minutes per mouse. Learning criteria was opening the door within 60 seconds for 3 days successively. As mice could be affected by each other, which could interfere with their empathy-like behavior, we reduced contact between the mice during the behavioral assessment. After empathy test, anxiety levels were evaluated with elevated plus maze and open field tests. All behavioral experiments were conducted in the morning between 9:00 and 13:00 hours.

### Open field test

Open field is a square area with 1-m borders, surrounded by walls 50 cm in height. Open field chamber was placed in a soundproof room with controlled illumination (100l×). After placing each mouse in the center of the chamber, its locomotor activity was recorded with a video camera mounted 2.5 m above the chamber for 5 minutes. Open field was divided into 16 equal squares (4 central, 12 peripheral). The distance traveled by the mice and the time spent in different parts of the open field were calculated. Time spent in the central part was considered as an index of anxiolysis (21).

### Elevated plus maze

The elevated plus maze is a plus-shaped apparatus commonly used to evaluate anxiety in rodents based on the rodents’ aversive behavior to open spaces. Maze was 50 cm above the floor level and consisted of 4 arms, two of which were open and two closed, with walls 40 cm in height. Mice were placed in the center of the apparatus facing the open arm. The locomotor activity of mice was recorded for 5 minutes. Time spent in the closed and open arms and total number of entries into the closed and open arms were calculated. Time spent in the open arms of equipment was considered as an index of anxiolysis.

### Biochemical analysis

Thoracotomy was performed under carbon dioxide anesthesia, and blood sample was obtained from the ventricle by cardiac puncture, followed by extraction of brain tissues. Tissue samples (blood and brain) were stored in the laboratory refrigerator (-85°C) until analysis. Mouse Oxytocin ELISA kit (catalog no: E-EL-0029, Elabscience, Wuhan, China) was used for oxytocin analysis. Assay sensitivity was determined to be 9.38 pg/mL and the detection range to be 15.63-1000pg/mL.

### Statistical evaluation

SPSS software for Windows, version 11.0 (SPSS, Chicago, IL) was used for statistical analysis. GLM-repeated measure post-hoc Bonferroni was used to analyze differences in empathy-learning period. Mann-Whitney U test was used to analyze differences between the groups. Pearson correlation analysis was used to calculate correlations between the groups. Results were presented as mean ± standard deviation. Because multiple comparisons were performed to prevent an alpha (type 1) error, (a/n=0.05/5), p<0.01 was considered statistically significant (22).

## RESULTS

Mean running distance for the exercise group in the running wheel cages was 2.4±0.3 km/day, and the running time was 143±5.18 min/day.

**Figure 2:**
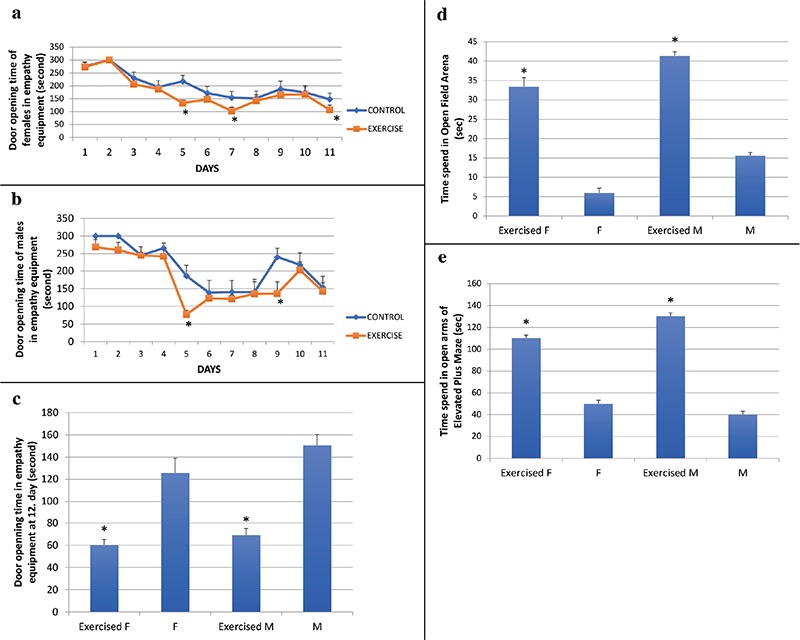
Behavioral test results; Comparison of exercise and control groups’ door-opening time of the empathy test equipment (lower time periods indicate more empathy like behavior) during the total of 12 days – female rats (a), Comparison of exercise and control groups’ door-opening time of the empathy test equipment during the total of 12 days – male rats (b), Comparison of exercise and control groups’ door-opening time of the empathy test equipment on day 12 (c), Comparison of exercise and control groups’ time spent in the open field arena (higher time periods indicate lower anxiety behavior) (d), Comparison of exercise and control groups’ time spent in the open arms of elevated plus maze (higher time periods indicate lower anxiety behavior) (e). F: females; M: males; *p<0.05 compared to same sex control group

In Helping Behavior test equipment, the mean door-opening duration progressively decreased with time in all the groups (p<0.0001) (Figure 2a, b). Exercised groups opened the door quicker than the control groups in the empathy-learning period (5^th^ day, exercised females 186±103.9 and control females 217±101.2, p=0.027; 7^th^ day, exercised females 141±48.56 and control females 153.89±102.9, p=0.001; 5^th^ day exercised males 76.7±42.9 and control males 132.9±50.1, p=0.01; and 9^th^ day, exercised males 136.4±123.6 and control males 214.3±65.6, p=0.001) (Figure 2a, b). On the test day (day 12), door-opening time was observed to decrease in the exercised mice (exercised females 125.8±45.4; control females 150.6±40.8; exercised males 60.7±16.9; control males 69.3±22.1; both genders p=0.0001) (Figure 2c).

Times spent in the center of the open field chamber were higher in exercised groups (exercised females 33.4±5.7; control females 6.1±2.8; exercised males 41.4±2.5; control males 15.6±1.9, both genders p=0.002) (Figure 2d). There was no difference between the males and females.

In the elevated plus maze test, the exercise groups spent more time in the open arms when compared to the same gender of control groups (exercised females 80.4±19.2; control females 50.1±18.9, p=0.004; exercised males 130.5±32.6; control males 40.1±5.9, p=0.0001) (Figure 2e).

**Figure 3:**
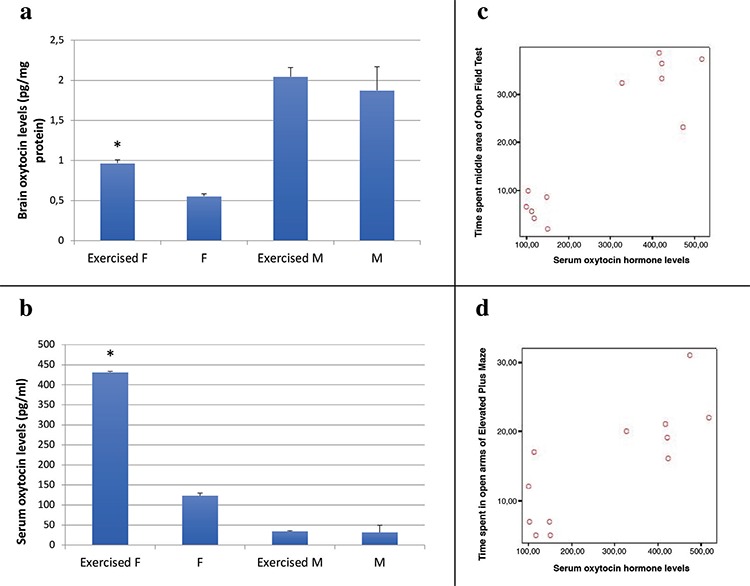
Biochemical analysis and correlation results; Comparison of exercise and control groups’ brain tissue (a) and serum (b) oxytocin levels F: females; M: males; *p<0.05 compared to same sex control group, Scatterplot graphics of the relationship between oxytocin and the activity in the middle area of the open field test (c) and the time spent in the open arms of the elevated plus maze test (d).

The brain and serum oxytocin levels were found to be higher in exercised female mice (Brain; exercised females 0.87±0.13; control females 0.55±0.09, p=0.0001; exercised males 2.44±0.39; control males 1.87±1.02, p=0.039; Serum; exercised females 431.49±60.76; control females 123.38±21.15, p=0.0001; exercised males 34.49±4.79; control males 31.59±5.88, p>0.05) (Figure 3a, b), whereas, statistically, no significant difference was observed in males (p>0.05).

In females, a strong negative correlation was found between the serum oxytocin levels and door-opening time of helping behavior equipment (r=-0.702, p=0.0001), and a moderate negative correlation was found between the brain oxytocin levels and door-opening time of helping behavior equipment (r=-0.430, p=0.036). However, there was no correlation between both the brain and serum oxytocin levels and empathy behavior in males.

In females, there were very strong positive correlations between the activity in the central area of open field and both brain and serum oxytocin levels (brain oxytocin, r=0.815, p=0.0001; serum oxytocin, r=0.914, p=0.0001) (Figure 3c). Serum oxytocin levels were strongly correlated with activity in the central area of open field in males (r=0.871, p=0.0001) and activity in the open area of elevated plus maze test in both genders (r=0.813, p<0.0001 in females; r=0.909, p=0.0001 in males) (Figure 3d). Also, brain oxytocin levels were strongly correlated with activity in the open area of elevated plus maze test in females (r=0.558, p<0.005).

## DISCUSSION

Voluntary physical activity decreased anxiety and increased empathy-like behavior in both male and female mice in the present study. These results were related with increased oxytocin levels in females but not in males. To our knowledge, this is the first study that examines the relationship between physical activity and oxytocin, anxiety, and empathy-like behavior.

Voluntary exercise is recommended in stress-related disorders, such as anxiety disorders. Although conflicting results have been reported about the relationship between anxiety and chronic voluntary exercise (23,24), chronic voluntary exercise was found to reduce anxiety in our previous studies (2). Moreover, in this study, exercised mice of both genders showed reduced anxiety in behavioral tests. Empathy is a psychological identification with the feelings, thoughts, or attitudes of another. It also forms the basis of the feeling of interest, warmth, and closeness to those in difficult situations. Empathy-related helping behavior (prosocial behaviors) involves helping others in a difficult situation (13). Empathy-related behaviors in sports consist of certain behaviors, such as helping a falling opponent, encouraging teammates, etc. (25). There is a great amount of research on the relationship between empathy and prosocial behaviors, stating that in the lack of empathy, prosocial/helping behavior is not observed. A significant positive relationship between sportsmanship orientation (prosocial/helping behavior) and empathy has been previously reported (25,26). Differing results of empathy-like behavior during a sporting event have been observed. Empathy toward an opponent may conflict with the “achievement goal”, whereas empathizing with a teammate may hinder focusing on personal performance (27). In some cases, coaches intentionally may not want their athletes to worry about their opponents (28). Although there is research on the effects of empathic behavior during a sporting event, the effect of regular physical activity on social empathic behaviors is not known. In our study, we found that exercised mice displayed higher levels of empathy than the controls.

We found that the brain and serum oxytocin levels post exercise were higher in the exercised females but unchanged in males. Exercise increases oxytocin release from nucleus tractus solitarius. Oxytocin is important in autonomic changes resulting from voluntary exercise that stimulates the parasympathetic nervous system, reducing the cardiac tone through nervus vagus and limiting exercise-induced tachycardia (29). There are a few studies reporting the effects of exercise on oxytocin levels. Martins et al. (30) reported that chronic treadmill exercise increased oxytocin and oxytocin receptor levels in the paraventricular nucleus of hypothalamus and its projection to the dorsal brain stem in male rats. Kim et al. (31) reported that the running wheel exercise reversed depression and increased oxytocin and oxytocin receptor levels in the basolateral amygdala in male mice. Interestingly, chronic resistance exercise was reported to reduce oxytocin levels in the paraventricular neurons of the hypothalamus in male rats (32). In addition, Bakos et al. (33) showed that 3 weeks of running wheel exercise decreased pituitary and serum oxytocin levels in male Sprague Dawley and Lewis rats. In one of the studies on humans, Hew-Butler et al. (34) showed that whereas blood oxytocin levels increased in both ultramarathon runners and high intensity exercising groups, they did not change in the steady-state control group. Their subjects consisted of both males and females. Most of the studies report significantly higher levels of oxytocin in female species than in males; however, to our knowledge, this is the first study to compare oxytocin levels in regular voluntary exercised subjects.

Moreover, in our study, high blood and brain oxytocin levels positively correlated with low anxiety levels and increased empathy-like behavior levels in females.

Our findings suggest that voluntary physical activity decreases anxiety and increases empathy-like behavior in both male and female mice. In females, blood and brain oxytocin levels were also found to be significantly higher than those in controls and were correlated with behavioral results, whereas, there was no change in oxytocin levels of males. These results suggest that brain and blood oxytocin levels are related to anxiety and empathy-like behaviors only in exercised females, and other mechanisms have a role in the positive effects of exercise on anxiety and empathy-like behaviors in males. Further investigation is required on the mechanisms of exercise effect on anxiety and empathic brain pathways in males.
